# Phosphorus pentasulfide mediated conversion of organic thiocyanates to thiols

**DOI:** 10.3762/bjoc.13.117

**Published:** 2017-06-20

**Authors:** Chandra Kant Maurya, Avik Mazumder, Pradeep Kumar Gupta

**Affiliations:** 1 Synthetic Chemistry Division, Defence Research & Development Establishment (DRDE), Jhansi Road, Gwalior (MP)-474002, India; 2 Vertox Division, Defence Research & Development Establishment (DRDE), Jhansi Road, Gwalior (MP)-474002, India

**Keywords:** dithiocarbamate, phosphorus pentasulfide, thiocyanate, thiol, toluene

## Abstract

In this paper we report an efficient and mild procedure for the conversion of organic thiocyanates to thiols in the presence of phosphorus pentasulfide (P_2_S_5_) in refluxing toluene. The method avoids the use of expensive and hazardous transition metals and harsh reducing agents, as required by reported methods, and provides an attractive alternative to the existing methods for the conversion of organic thiocyanates to thiols.

## Introduction

**Scheme 1:**
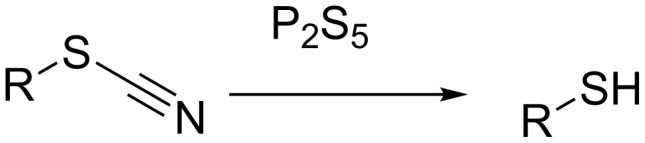
Conversion of organic thiocyanates to thiols.

Thiols constitute an important group of sulfur-containing compounds. They have a specific odour and often used as gas odorants in many industrial applications [1–2]. They occur as flavouring compounds in several fruits and spices and are found in a variety of enzymes at their active sites [3]. Thiols are produced by the wood-pulping industry, manure & sewer systems and by the breakdown of sulfur-containing amino acids and lignin [4–7]. In laboratory, thiols can be synthesized from alcohols [8–9], alkyl halides [10], alkenes [11] and through reductive cleavage of organic thiocyanates (or simply thiocyanates hereafter) by means of alkali metals–ammonia, Zn–HCl, catalytic hydrogenation (H_2_–molybdenum disulfide) and metal hydrides like LAH [12–17]. These methods, however, suffer from disadvantages like slow reaction rates, poor product yields, involvement of expensive and harsh reagents [16] and predominant side reactions leading to the formation of monosulfides which still leave scope for further investigations in this area. With this context, we, herein, describe an alternative method, devoid of shortcomings of the reported methods, for the conversion of thiocyanates to the corresponding thiols mediated by P_2_S_5_ under non-reductive conditions (Scheme 1). Phosphorus pentasulfide (P_2_S_5_), a commercially available reagent, has widely been employed in organic synthesis for numerous applications [18]. The synthetic protocol described in this paper makes use of this reagent to provide an efficient and single step procedure for the conversion of thiocyanates to the corresponding thiols.

## Results and Discussion

Initially, for reaction condition optimizations, benzyl thiocyanate was chosen as model substrate and was reacted with P_2_S_5_ in different organic solvents (Table 1). Although, the reaction proceeded in solvents including benzene, toluene, THF, dichloromethane, the best results in terms of reaction time and yields were obtained in toluene at refluxing temperature. The optimization studies also revealed that a substoichiometric amount of reagent and the reaction at room temperature resulted in a low product yield or incomplete reaction. Most importantly, the reaction furnished only thiols as the final product with no disulfide formation. Interestingly, no similar reaction of thiocyanates was observed with other thionating agents like Lawesson’s reagent and PSCl_3_ and the reactant was recovered quantitatively in those cases.

**Table 1:** Effect of reaction medium and reagents on conversion of thiocyanates to thiols.^a^

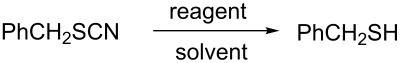				

Entry	Solvent	Reagent	Time (h)	Yield (%)

1	THF	P_2_S_5_	2.0^b^	45
2	DCM	P_2_S_5_	2.0^c^	40
3	benzene	P_2_S_5_	2.0^b^	60
4	toluene	P_2_S_5_	1.5^b^	85
5	THF	Lawesson’s reagent	4.0^b^	–
6	THF	PSCl_3_	4.0^b^	–

^a^Benzyl thiocyanate (10 mmol), reagent (10 mmol), solvent (25 mL); ^b^reflux temperature; ^c^room temperature.

In order to further study the scope and limitations of the method, different thiocyanates were treated with P_2_S_5_ in refluxing toluene to get the corresponding thiols in good to moderate yield in short reaction time (Table 2). The thiocyanate substrates were prepared by the reaction of alkyl halide with potassium thiocyanate in refluxing propylene glycol [19]. Simple short chain thiocyanates were found to react rapidly to give the corresponding thiols. In comparison to the alkyl substrates, the benzyl derivative reacted sluggishly possibly due to electronic effects which was further evident by the longer reaction time required by 3-phenoxybenzyl thiocyanate due to the presence of the bulkier 3-phenoxy group (Table 2, entry 15). In the case of benzyl thiocyanates, the presence of an electron donating methoxy group on the phenyl ring (Table 2, entry 14) caused a reaction rate acceleration while the presence of electron withdrawing halogens on the phenyl ring diminished the rate of reaction. It was interesting to note that in this reaction, product yields were found to be less susceptible to substituent effects in comparison to the reaction rates. One of the noticeable features of the present method is the tolerance of reducible functional groups like a nitro group and a triple bond in the reaction which cannot be used in the reported methods.

**Table 2:** Conversion of different thiocyanates to thiols with P_2_S_5_ in refluxing toluene^a^.

Entry	Reactant	Time (h)	Product*^b^*	Yield (%)

1	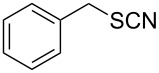	1.5	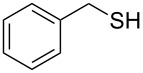 **1**	85
2	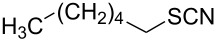	1.0	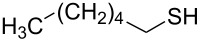 **2**	93
3	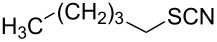	1.5	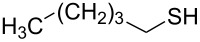 **3**	87
4	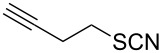	1.2	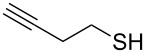 **4**	75
5	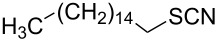	2.0	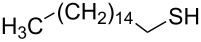 **5**	81
6	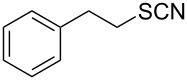	2.0	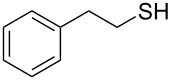 **6**	80
7	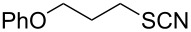	2.0	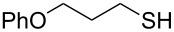 **7**	85
8	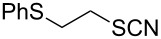	2.5	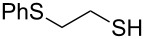 **8**	89
9	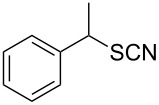	3.0	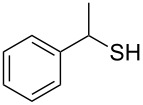 **9**	83
10	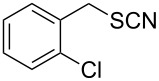	3.0	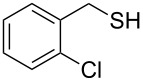 **10**	79
11	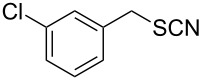	3.0	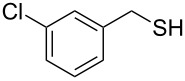 **11**	81
12	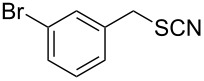	3.0	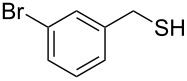 **12**	78
13	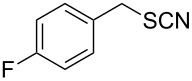	3.0	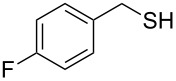 **13**	75
14	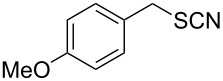	1.5	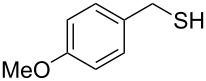 **14**	89
15	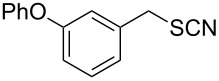	4.0	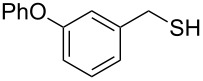 **15**	78
16	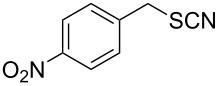	2.0	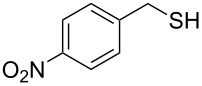 **16**	73

*^a^*Reaction conditions: thiocyanate (10 mmol), P_2_S_5_ (10 mmol), toluene (25 mL), reflux. ^b^Identification of all the products was carried out comparing them with authentic samples available commercially except entries 7, 8, 11, 15 which were characterized by spectral analysis.

**Scheme 2:**
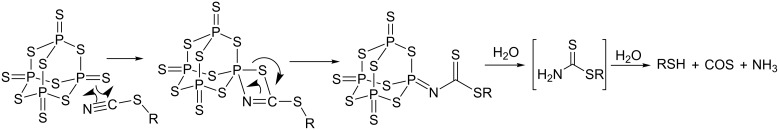
Hypothetical mechanism for conversion of thiocyanates to thiols mediated by phosphorus pentasulfide.

A hypothetical mechanism (Scheme 2) for this conversion was believed to involve, in analogy with the thionation of nitriles to thioamides [20], initial thionation of the thiocyanate functionality with P_2_S_5_ to give the corresponding dithiocarbamate derivative which, in presence of residual phosphoric acid of P_2_S_5_, decomposes to give the corresponding thiol, in analogy with the acidic hydrolysis of *S*-thiocarbamates [21]. Although, we were not able to isolate the *S*,*S*-dithiocarbamate intermediate from the reaction between thiocyanate and P_2_S_5_, its formation was indirectly confirmed by treating benzyl *S*-thiocarbamate, synthesized separately [22], with P_2_S_5_ under similar reaction conditions to give the corresponding thiol (80%). It could be believed that thionation of benzyl *S*-thiocarbamate led to the formation of benzyl *S*,*S*-dithiocarbamate which underwent acidic hydrolysis to give the corresponding thiol.

## Conclusion

In summary, the method described in this paper presents an efficient and direct route for the conversion of organic thiocyanates to the corresponding thiols. It further provides an indirect route for the conversion of alkyl halides and alcohols to the corresponding thiols through their thiocyanate derivatives. Unlike the reported methods, the present method works under non reductive conditions and eliminates the use of harsh and expensive reducing agents, as required by the reported methods. In this way, this method presents an attractive method for the preparation of thiols which, in addition, can be useful for the generation of a thiol functional group during a total synthesis.

## Experimental

**General experimental procedure**: In a three-neck round bottom flask, to a solution of thiocyanate (10 mmol) in toluene (25 mL), P_2_S_5_ (2.22 g, 10 mmol) was added and the resulting suspension was refluxed till complete consumption of the starting material (TLC). After the reaction was complete, the reaction mixture was quenched by careful addition of water (10 mL), extracted with ethyl acetate (3 × 10 mL), the organic phase was dried over sodium sulfate and evaporated under reduced pressure to get the crude product which was purified by flash chromatography (hexane–ethyl acetate) to get the pure thiol.

### 3-Phenoxypropylthiol (**7**)

Oil (1.43 g, 85%). IR (KBr, ν_max_): 3155, 3065, 2929, 2872, 2362, 1695, 1598, 1242 cm^−1^; ^1^H NMR (600 MHz, CDCl_3_) δ 7.30–7.27 (m, 2H), 6.96–6.89 (m, 3H), 4.06 (t, *J* = 12 Hz, 2H, OCH_2_), 3.42 (t, *J* = 12 Hz, 2H, SCH_2_), 2.21 (qn, *J* =12 Hz, 2H, CCH_2_), 1.28 (s, 1H, SH); ^13^C{^1^H} NMR (125 MHz, CDCl_3_) δ 158.66, 129.51, 120.91, 114.53, 65.98, 33.62, 28.77; EIMS (*m*/*z*): 51 (7), 65 (12), 66 (8), 74 (10), 75 (7), 77 (16), 94 (100), 95 (8), 168 (20); HRMS (ESI) *m*/*z*: [M + Na]^+^ calcd for C_9_H_12_OSNa, 191.0609; found, 191.0610.

### 2-(Phenylthio)ethanethiol (**8**)

Oil (1.51 g, 89%). IR (KBr, ν_max_): 3059, 2964, 2363, 1261, 1094, 1026 cm^−1^; ^1^H NMR (600 MHz, CDCl_3_) δ 7.30–7.28 (m, 2H), 7.23–7.14 (m, 3H), 3.11 (m, 2H, PhSCH_2_), 2.76 (m, 2H, SCH_2_), 1.18 (s, 1H, SH); ^13^C{^1^H} NMR (125 MHz, CDCl_3_) δ 133.97, 129.28, 128.91, 128.07, 128.03, 125.59, 36.64, 32.17; EIMS (*m*/*z*): 51 (16), 61 (23), 66 (8), 69 (12), 77 (17), 78 (8), 91 (5), 109 (24), 110 (100), 111 (8), 123 (28), 170 (26); HRMS (ESI) *m*/*z*: [M + Na]^+^ calcd for C_8_H_10_S_2_Na, 193.0224; found, 193.0225.

### 3-Chlorobenzylthiol (**11**)

Oil (1.28 g, 81%). IR (KBr, ν_max_): 2965, 2363, 1262, 1096, 1026 804 cm^−1^; ^1^H NMR (600 MHz, CDCl_3_) δ 7.25–7.17 (m, 2H), 7.13–7.04 (m, 3H), 3.49 (s, 2H, CH_2_), 1.18 (s, 1H, SH); ^13^C{^1^H} NMR (125 MHz, CDCl_3_) δ 136.26, 133.27, 128.77, 128.44, 126.67, 126.50, 41.60; EIMS (*m*/*z*): 63 (12), 75 (6), 89 (23), 99 (7), 125 (100), 127 (31), 158 (34), 160 (12); HRMS (ESI) *m*/*z*: [M + Na]^+^ calcd for C_7_H_7_ClSNa, 180.995; found, 180.9956.

### 3-Phenoxybenzylthiol (**15**)

Oil (1.68 g, 78%). IR (KBr, ν_max_): 3150, 2958, 2363, 1539, 1249, 1032 cm^−1^; ^1^H NMR (600 MHz, CDCl_3_) δ 7.34–7.24 (m, 3H), 7.10–6.87 (m, 6H), 3.87 (d, *J* = 12 Hz, 2H, CH_2_), 1.75 (t, *J* = 12 Hz, 1H, SH); ^13^C{^1^H} NMR (125 MHz, CDCl_3_) δ 157.50, 156.94, 143.11, 129.92, 129.76, 123.37, 122.77, 119.01, 118.34, 117.29, 28.70; EIMS (*m*/*z*): 51 (12), 77 (17), 89 (9), 91 (8), 153 (10), 168 (11), 181 (10), 183 (100), 184 (15), 216 (71), 217 (10); HRMS (ESI) *m*/*z*: [M + Na]^+^ calcd for C_13_H_12_OSNa, 239.0609; found, 239.0611.

## Supporting Information

File 1 Typical experimental procedure, ^1^H and ^13^C spectra of **7**, **8**, **11**, **15**.
