# Supplementary material for: Phosphorus pentasulfide mediated conversion of organic thiocyanates to thiols
Source: Beilstein J Org Chem. 2017 Jun 20;13:1184–8. doi: 10.3762/bjoc.13.117 (PMC5496577; doi:10.3762/bjoc.13.117)
Supplement: File 1 — Typical experimental procedure, 1H and 13C spectra of 7, 8, 11, 15. [file Beilstein_J_Org_Chem-13-1184-s001.pdf]

Supporting Information

for

Phosphorus pentasulfide mediated conversion of organic thiocyanates to thiols

Chandra Kant Maurya[1], Avik Mazumder[2] and Pradeep K Gupta[*1]

Address: [1]Synthetic Chemistry Division, Defence Research & Development Establishment (DRDE), Jhansi Road, Gwalior (MP)-474002, India and [2]Vertox Division, Defence Research & Development Establishment (DRDE), Jhansi Road, Gwalior (MP)-474002, India

Email: Pradeep Kumar Gupta - pkgupta@drde.drdo.in

*Corresponding author

Typical Experimental Procedure, ^{1}H and ^{13}C NMR spectra of compounds 7, 8, 11, 15

Table of Content:

1. General information S2

2. Typical experimental procedure S2

3. ^{1}H and ^{13}C NMR spectra of compounds 7, 8, 11, 15 S3

1. General Information

In all reactions were carried out with commercial reagents without further purification. Progress of reactions was monitored by TLC on pre-coated Merck silica gel plates (60F-254) and GC MS. Visualization of reactants and products was accomplished with UV light and alkaline $KMnO_4$. Column chromatography was performed with a Combiflash Companion automated (ISCO) flash chromatography system. The ^1H NMR and ^{13}C NMR spectra were run on a Bruker Avance 600 NMR Spectrometer with $CDCl_3$ as solvent and TMS as an internal standard. GC MS analysis was carried out Agilent 5973i Mass spectral Detector attached to Agilent 6890 Gas Chromatograph.

2 Typical experimental procedure for conversion of conversion of thiocyanates to thiols

In three neck round bottom flask, to a solution of thiocyanate (10 mmol) in toluene (25 mL), P_2S_5 (2.22 g, 10 mmol) was added and resulting suspension was refluxed till complete consumption of the starting material (TLC). After the reaction was complete, the reaction mixture was quenched by careful addition of water (10 ml), extracted with ethyl acetate (3 × 10 mL), the organic phase was dried over sodium sulfate and evaporated under reduced pressure to get the crude product which was purified by flash chromatography (hexane–ethyl acetate) to get the corresponding thiol.

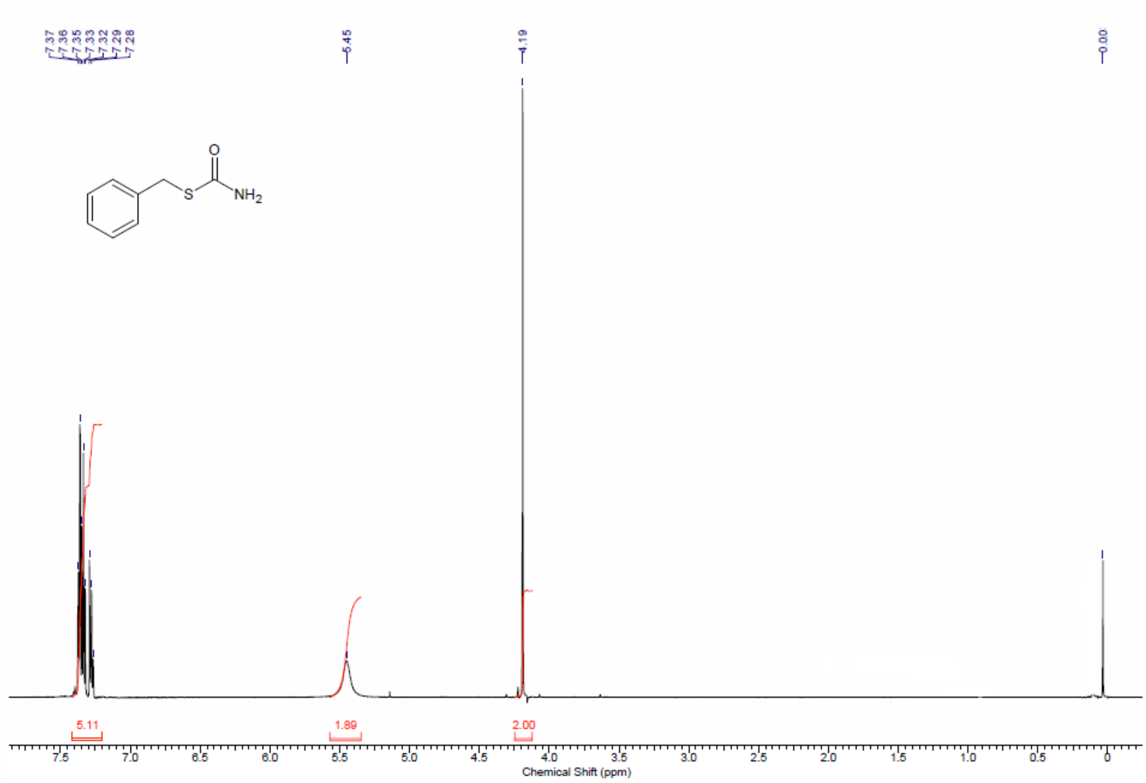
7.37
7.36
7.35
7.33
7.32
7.29
7.28
5.45
4.19
0.00
O
S
NH2
5.11
1.89
2.00
Chemical Shift (ppm)

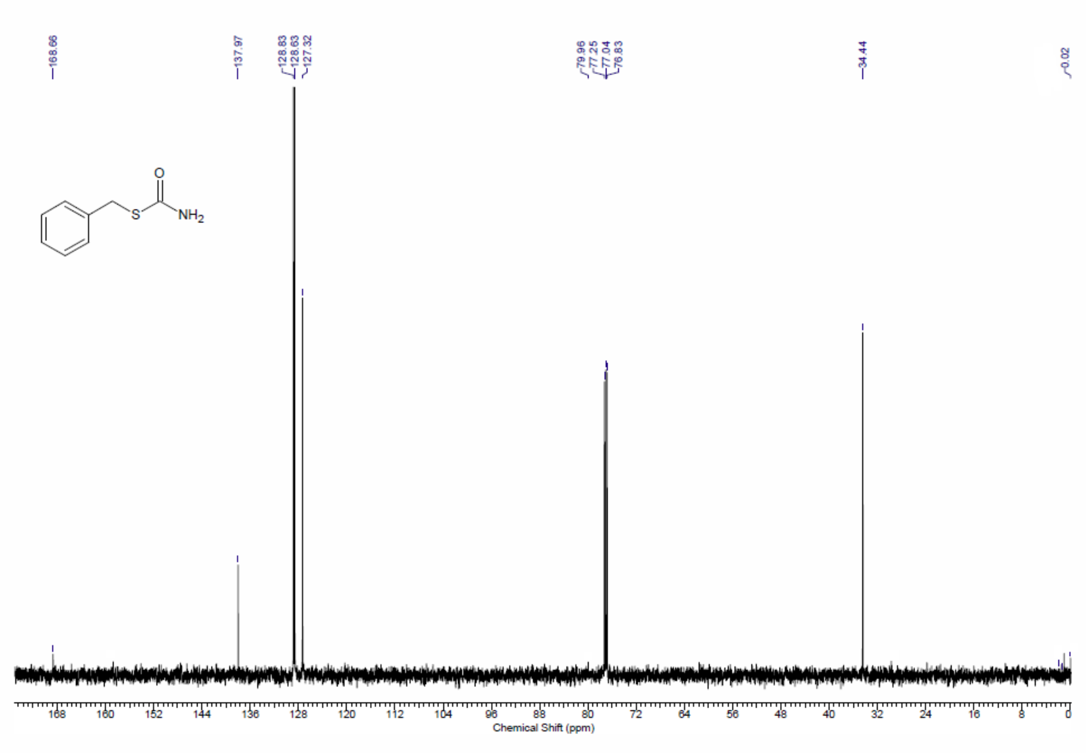
168.66
137.97
128.83
128.63
127.32
79.96
77.25
77.04
76.83
34.44
0.02
O
S
NH2
Chemical Shift (ppm)

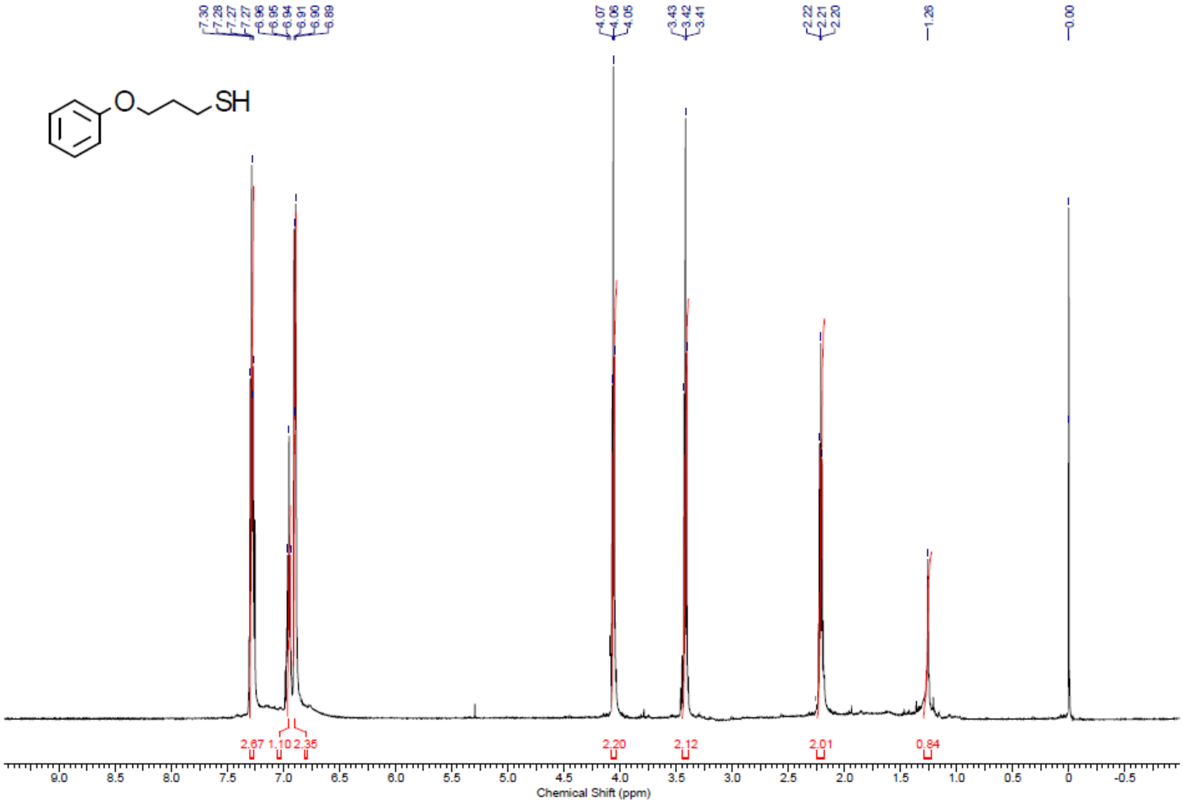
7.30
7.28
7.27
7.27
6.96
6.95
6.94
6.91
6.90
6.89
4.07
4.06
4.05
3.43
3.42
3.41
2.22
2.21
2.20
1.26
0.00
SH
O
2.67
1.10
2.35
2.20
2.12
2.01
0.84
Chemical Shift (ppm)

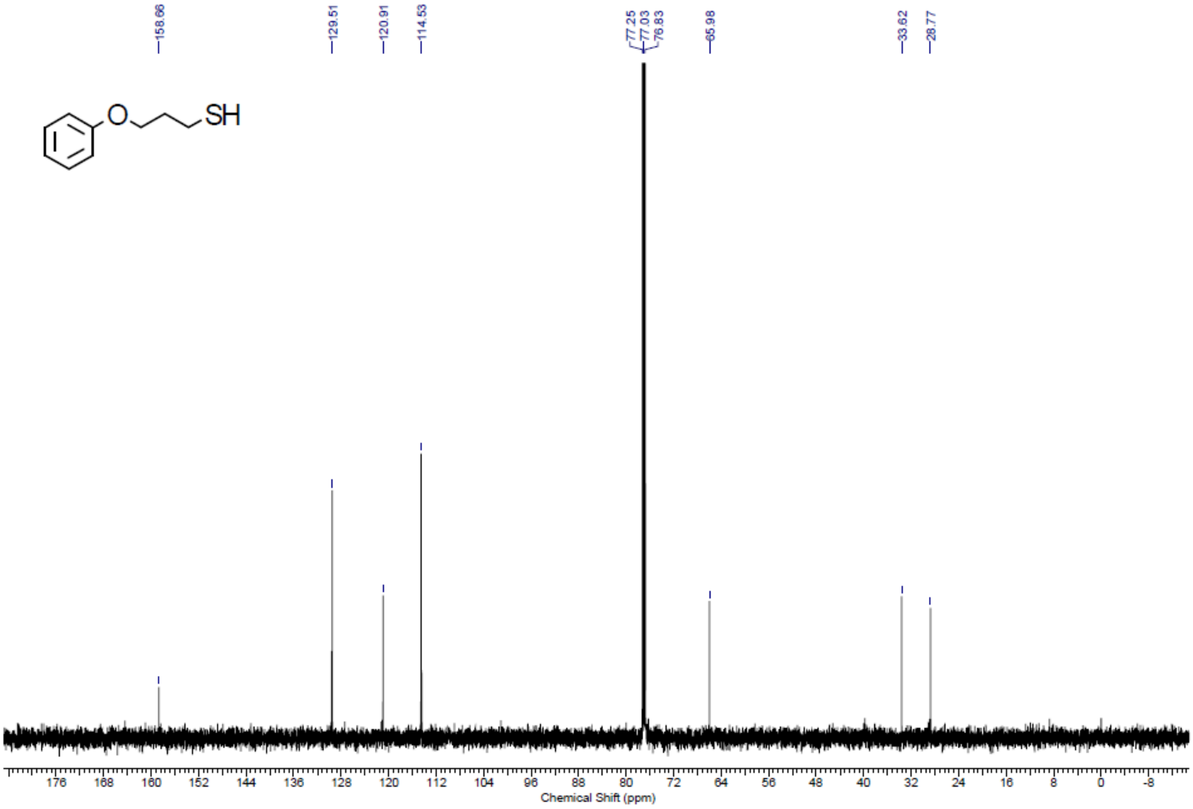
158.66
129.51
120.91
114.53
77.25
77.03
76.83
65.98
33.62
28.77
SH
O
Chemical Shift (ppm)

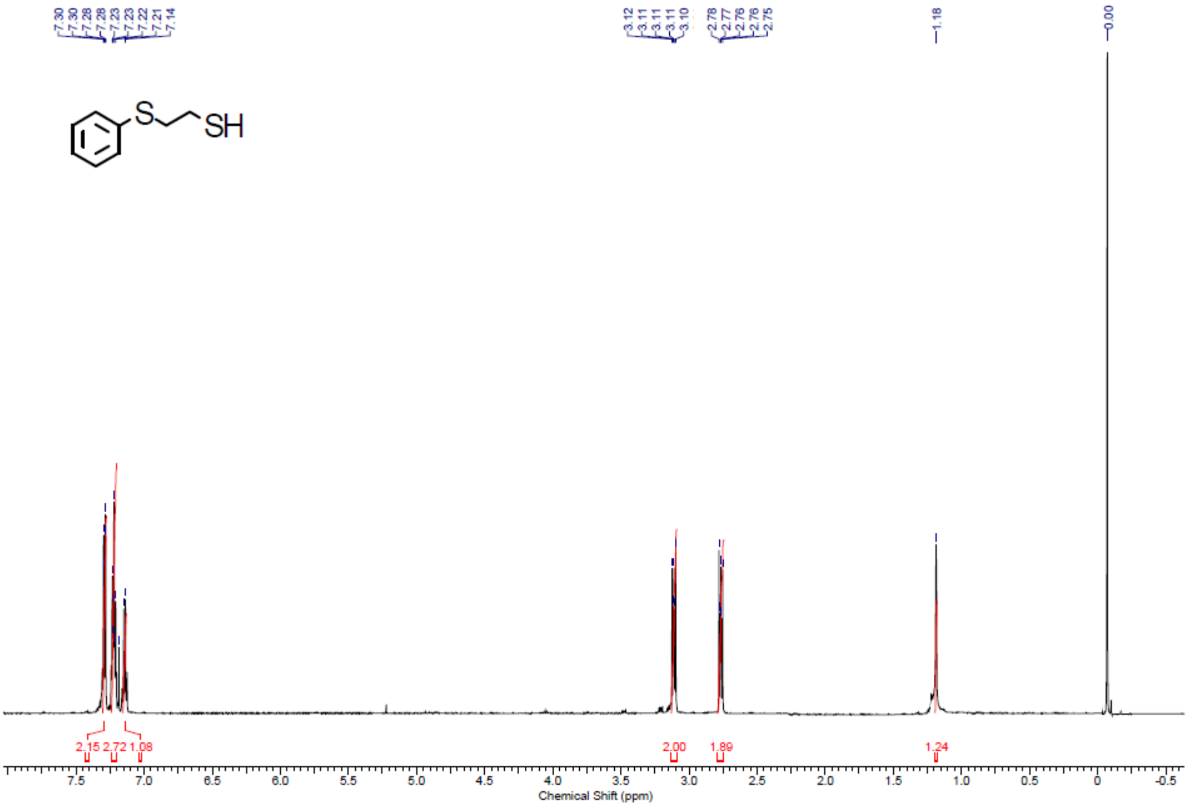
7.30 7.30 7.28 7.28 7.23 7.23 7.22 7.21 7.14
3.12 3.11 3.11 3.11 3.10
2.78 2.77 2.76 2.76 2.75
1.18
0.00
SH
S
2.15 2.72 1.08
2.00 1.89 1.24
Chemical Shift (ppm)

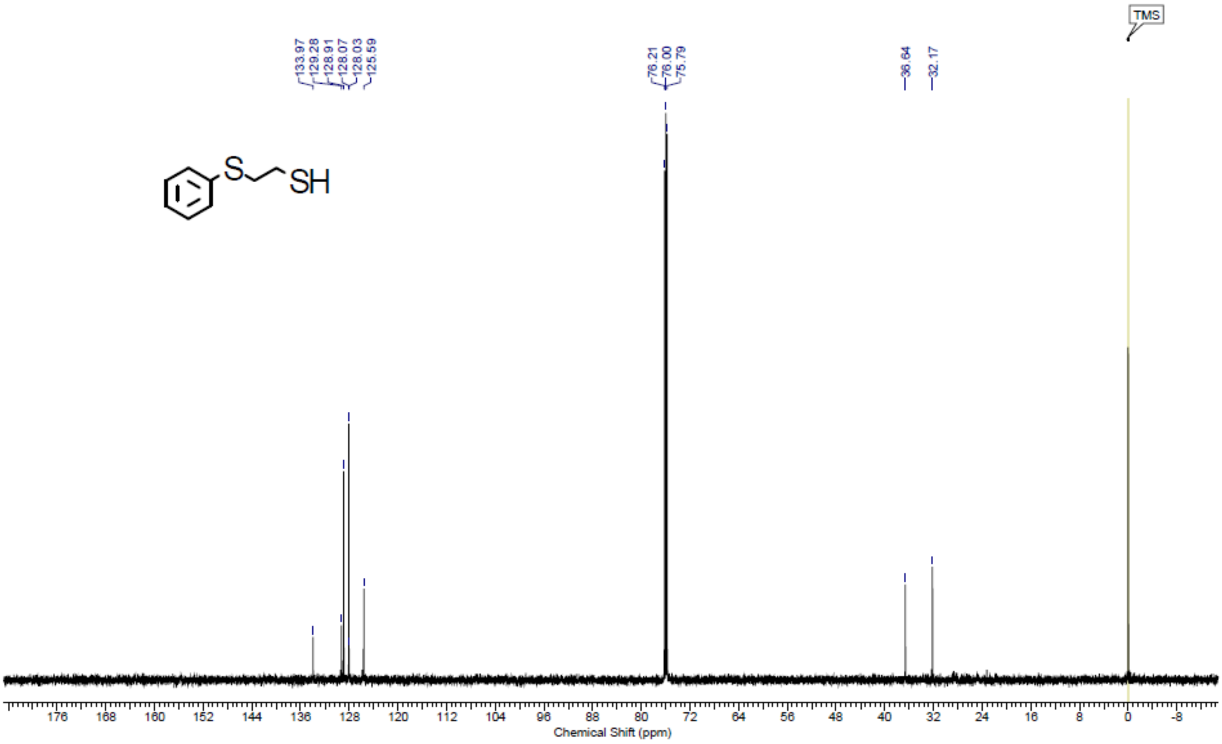
133.97 129.28 128.91 128.07 128.03 125.59
76.21 76.00 75.79
36.64 32.17
TMS
SH
S
Chemical Shift (ppm)

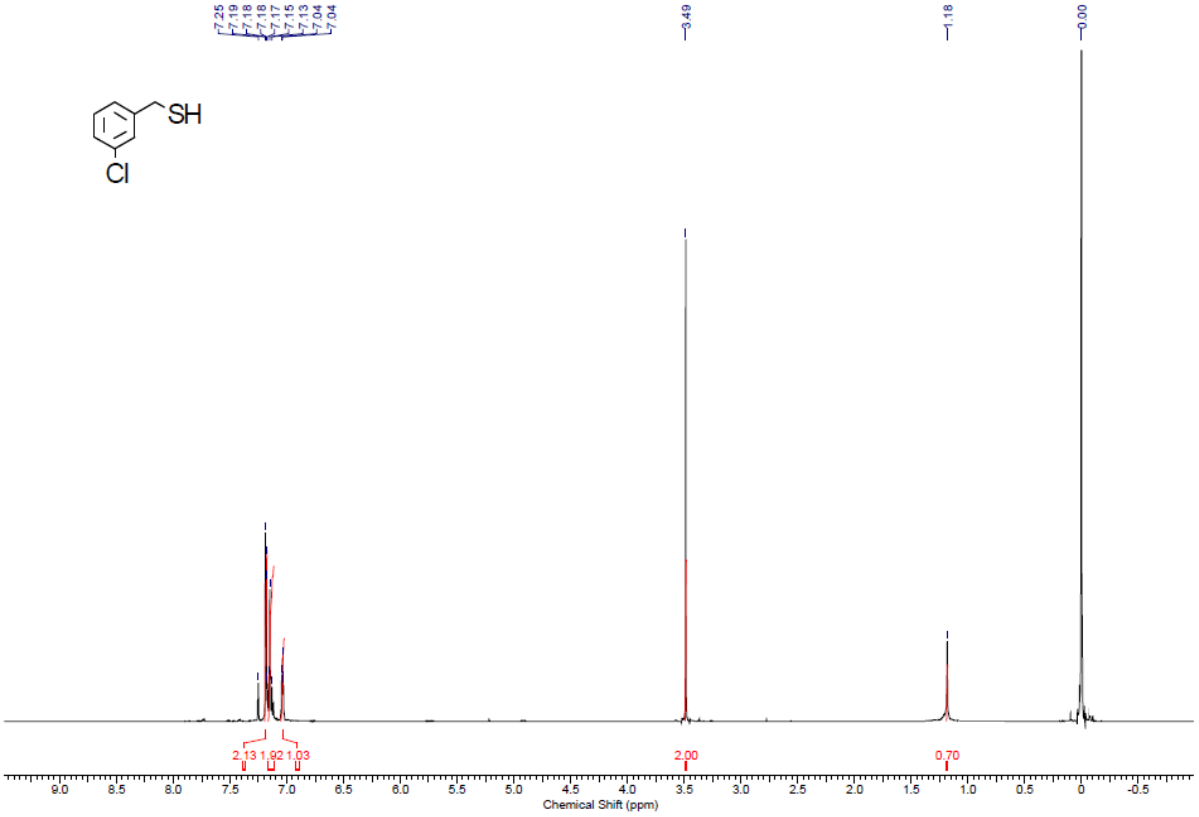
7.25
7.19
7.18
7.18
7.17
7.15
7.13
7.04
7.04
3.49
1.18
0.00
SH
Cl
2.13
1.92
1.03
2.00
0.70
Chemical Shift (ppm)

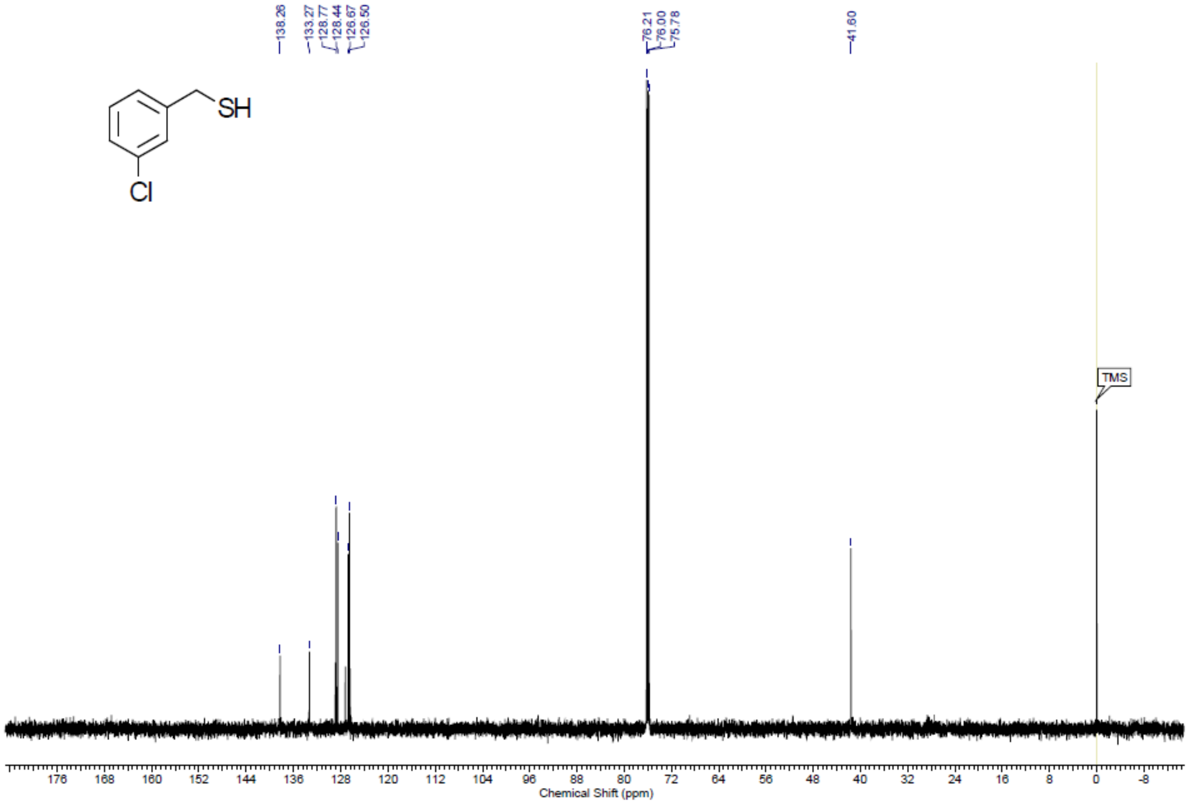
138.26
133.27
128.77
128.44
126.67
126.50
76.21
76.00
75.78
41.60
SH
Cl
TMS
Chemical Shift (ppm)

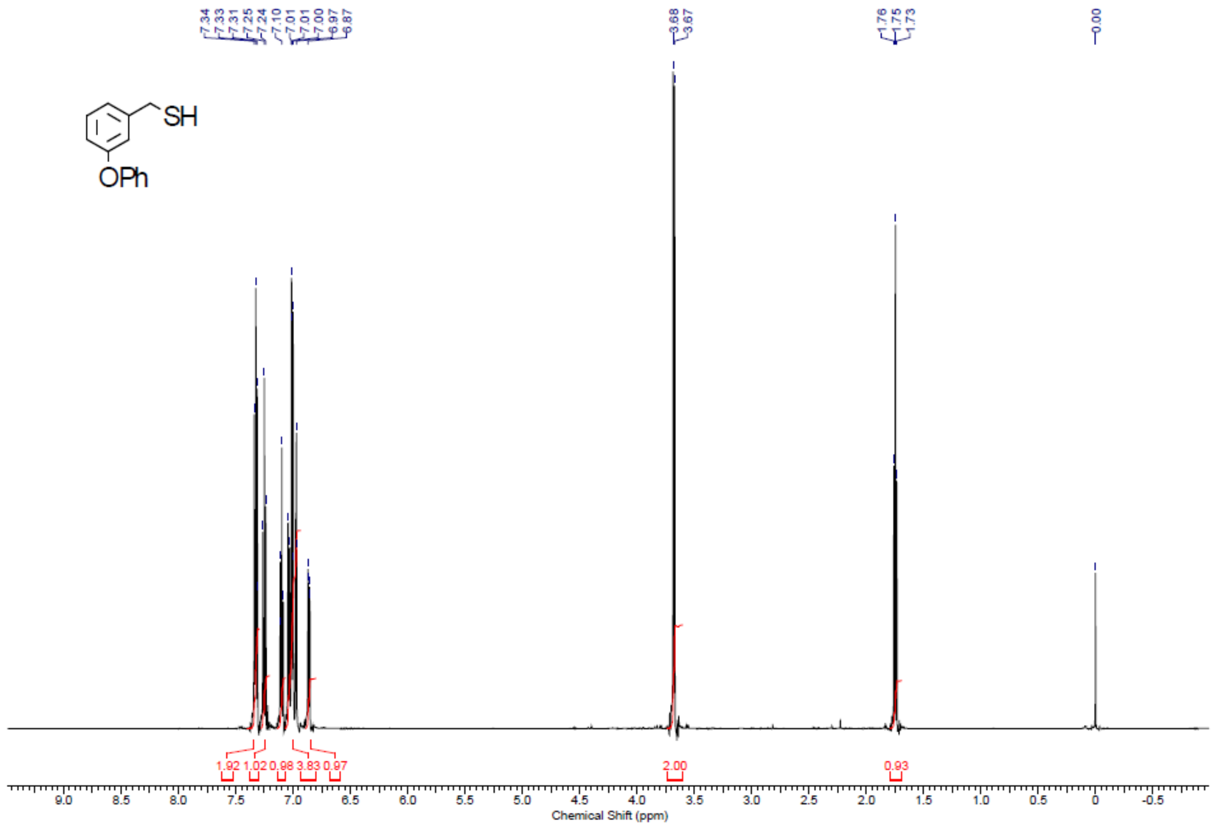
7.34
7.33
7.31
7.25
7.24
7.10
7.01
7.01
7.00
6.97
6.87
3.68
3.67
1.76
1.75
1.73
0.00
SH
OPh
1.92
1.02
0.98
3.83
0.97
2.00
0.93
Chemical Shift (ppm)

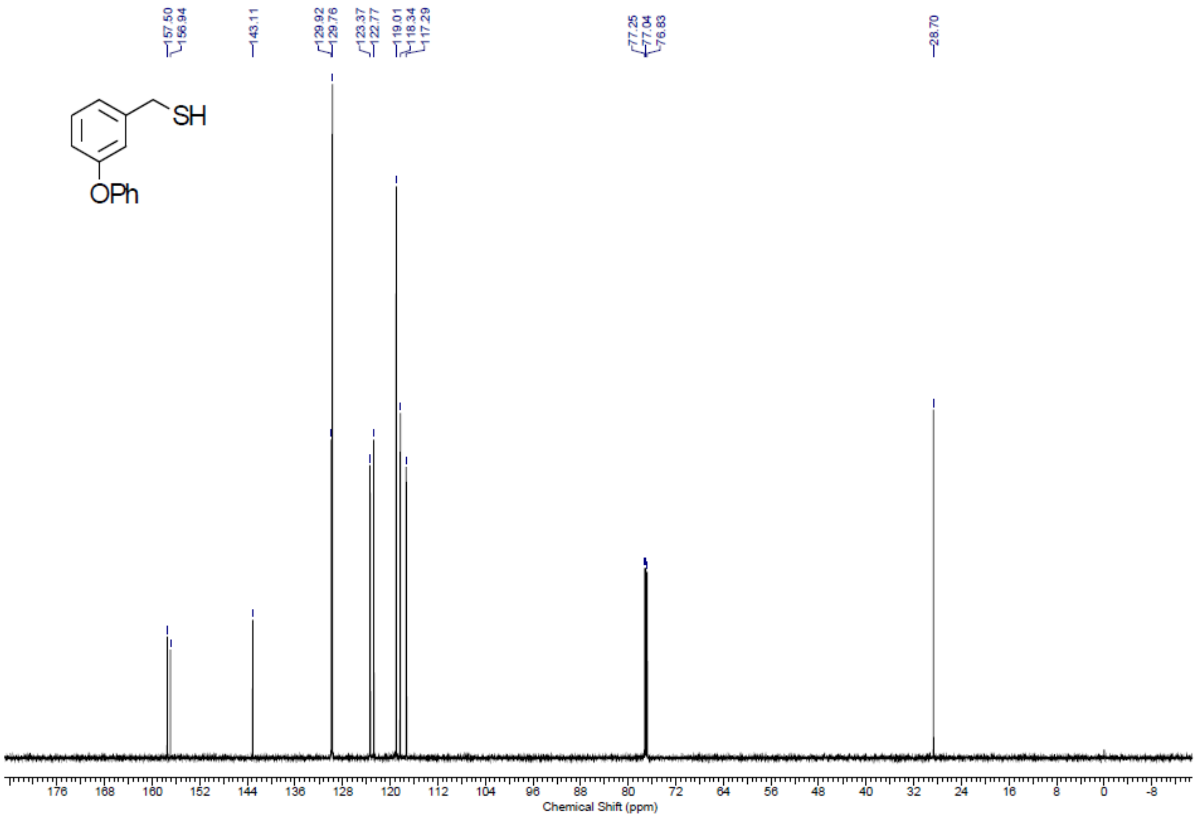
157.50
156.94
143.11
129.92
129.76
123.37
122.77
119.01
118.34
117.29
77.25
77.04
76.83
28.70
SH
OPh
Chemical Shift (ppm)
